# What we do not know about cerebellar systems neuroscience

**DOI:** 10.3389/fnsys.2014.00227

**Published:** 2014-12-18

**Authors:** Jan Voogd

**Affiliations:** Department of Neuroscience, Erasmus Medical Center RotterdamRotterdam, Netherlands

**Keywords:** cerebellar modules, mossy fiber systems, climbing fiber systems, purkinje cell zones, cerebro-cerebellar relations

## Abstract

Our knowledge of the modular organization of the cerebellum and the sphere of influence of these modules still presents large gaps. Here I will review these gaps against our present anatomical and physiological knowledge of these systems.

## Introduction

Advances in our knowledge of the functional anatomy of the cerebellum have raised new questions. In this review I will draw attention to some of, what I consider, the main flaws in our knowledge of the anatomy and physiology of cerebellar modules and their connections.

My main questions are:

1. The anatomical organization of the cerebellum is based on modules, defined by their projection to a specific cerebellar or vestibular nucleus, their climbing fiber input and the physiological and neurochemical properties of their Purkinje cells. Why do the modules in motor regions of the cerebellum alternate between those connected with motor pathways and those connected with wide regions of the cerebral cortex?
2. The cerebello-rubral pathway, the cerebello-thalamo-cortical projections and the corticorubral-olivary climbing fiber system seem to be organized as closed loops. What is the function of these loops and of the convergence of cortical and cerebellar nuclear input to the parvocellular red nucleus and other intercallated nuclei at the meso-diencephalic junction?
3. Which are the tractable behaviors with which to evaluate the hypothesis that each Purkinje cell zone constitutes a basic functional unit of the cerebellum (Simpson, 2011)?
4. What are the topographical and functional relations between mossy and climbing fibers in the cerebellar cortex?
5. Are mossy and climbing fiber pathways organized according to the same anatomical principles?
6. What are the topographical interrelations of different mossy fiber system in the cerebellum?

## The modular organization of the cerebellum

**Figure 1:**
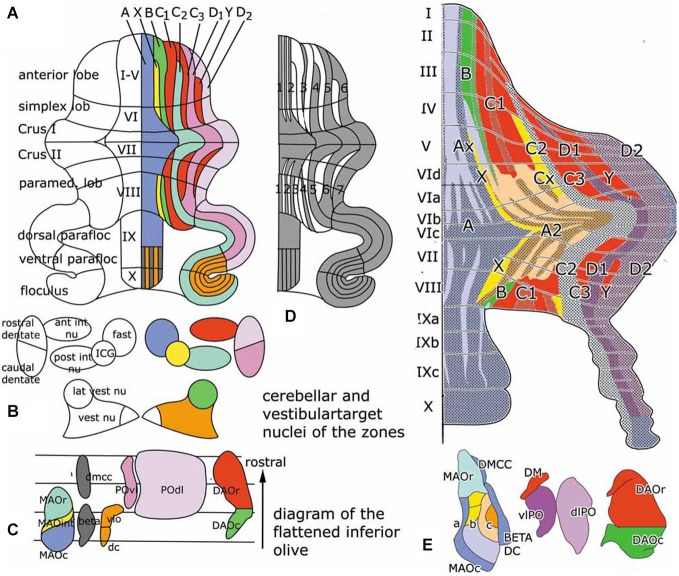
**(A)** Diagram of the flattened cerebellum of the rat showing the Purkinje cell zones **(A–D)**. The same color-code is used for the Purkinje cell zones in panels **(A)** and **(E)**, for the target nuclei of the zones in panel **(B)** and in the flattened map of the inferior olive, with the subnuclei that give rise to climbing fibers innervating the different zones in panel **(C)**. **(D)** Diagram of the distribution of zebrin-positive and –negative Purkinje cells. Zebrin-positive bands are numbered 1–7. **(E)** Sugihara and Shinoda (2004) diagram of the zebrin-positive and negative Purkinje cell zones. The main zones are indicated with the same colors as in panel **(A)** Redrawn from Sugihara and Shinoda (2004). Abbreviations: ant int nu, anterior interposed nucleus; DAOc/r, caudal/rostral dorsal accessory olive; dc, dorsal cap; DMCC, dorsomedial cell column; a, b, c, subnuclei a,b,c of caudal medial accessory olive; fast, fastigial nucleus; ICG, interstitial cell groups; lat vest nu, lateral vestibular nucleus; MAOr/int/c, caudal/intermediate/rostral subnucleus of the medial accessory olive; POdl, dorsal lamina principal olive; post int nu, posterior interposed nucleus; POvl, ventral lamina principal olive; vest nu, vestibular nuclei; vlo, ventrolateral outgrowth.

The cerebellum is known to be organized in a modular fashion. Cerebellar modules consist of one or more Purkinje cell zones that project to a particular cerebellar or vestibular nucleus, their climbing fiber input from a subdivision of the contralateral inferior olive with a collateral projection to the cerebellar target nucleus and reciprocal connections of this target nucleus with the contralateral inferior olive. Seven to nine of these modules originally were distinguished on both sides of the cerebellum in carnivores, rodents and primates (Figures 1A–C).

In rodents Purkinje cells that react with a Purkinje cell-specific antibody known as Zebrin, are distributed in a pattern of alternating zebrin-positive bands separated ny zebrin-.negative bands (Hawkes and Leclerc, 1987). More recently it was found that the zebrin pattern is congruent with the zonal organization of its corticonuclear and afferent climbing fiber projections (compare Figure 1, panels **(A)** and **(D)**). Purkinje cell zones, therefore, are either zebrin-positive or negative (Voogd et al., 2003; Sugihara and Shinoda, 2004). The zebrin signature stands for the distribution in these neurons of many different neuroactive substances, such as glutamate transporters and cytochrome oxidase (Apps and Hawkes, 2009). Moreover, zebrin-positive and negative Purkinje cells differ in their development, their physiological properties and the organization of their climbing fiber input from the periphery or the cerebral cortex. Zebrin-negative Purkinje cells are born later than the zebrin-positive ones and, in monkeys at least, reach the meningeal surface at a later stage. The medio-lateral compartmentation of the Purkinje cells, therefore, is determined at a very early stage of cerebellar development (Namba et al., 2011; Voogd, 2012). Purkinje cells of the mouse and rat zebrin-positive modules fire at a lower rate than Purkinje cells of zebrin-negative modules (Xiao et al., 2014; Zhou et al., 2014).

Zebrin negative zones occur in anterior and posterior regions of the rodent cerebellar hemisphere, in the anterior lobe and the simplex lobule, and in the paramedian lobule. These regions are considered as the “motor” regions of the cerebellar hemisphere because they were found to receive input from the primary motor cortex and to be involved in movement in several fMRI studies (Stoodley and Scmahmann, 2009). In rodents the zebrin-negative zones C1, C3 and Y alternate with the zebrin-positive zones C2, D1 and D2 (Figures 1A,D). The circuitry of the zebrin-negative zones typically is part of the motor system (Voogd and Ruigrok, 2004). The zebrin-positive zones maintain connections with wide areas of the cerebral cortex.

**Figure 2:**
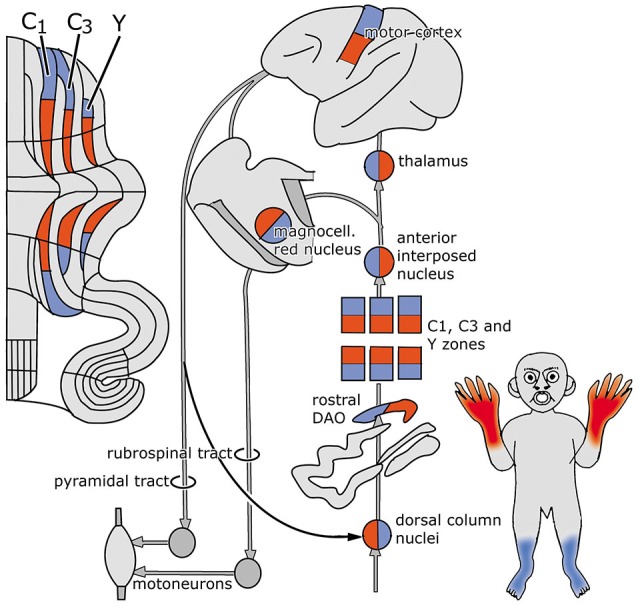
**Circuitry of the zebrin-negative C1, C3 and Y zones. Abbreviation: DAO, dorsal accessory olive**.

Figure 2 is based on the situation in the cat, where the C1, C3 and Y zones receive topically organized somatosensory climbing fiber input from the rostral dorsal accessory olive (DAO). The C1 and C3 zones also receive short-latency somatotopically arranged climbing fiber input from the contralateral posterior sigmoid gyrus (area 4γ: the motor cortex), relayed by the dorsal column nuclei. Long-latency climbing fiber input to these zones and climbing fiber input from wide areas of the cerebral cortex, including parietal and sensory areas to the C2 and D1 zones is not relayed by the dorsal column nuclei (Andersson and Nyquist, 1983; Andersson, 1984). The parvocellar red nucleus and other nuclei at the mesodiencephalic border my serve as links in these cortico-cerebellar pathways (Saint-Cyr, 1987).

The C1, C3 and Y zones project to the anterior interposed nucleus, where their somatopical maps converge upon a single map (Apps and Garwicz, 2000). This nucleus projects to the magnocellular red nucleus (Gibson et al., 1987) and, via the thalamus, to the motor and possibly to the premotor areas of the cerebral cortex (Kievit, 1979; Jörntell and Ekerot, 1999). These cortical areas give rise to the pyramidal tract that emits collaterals to the magnocellular nucleus (Catsman-Berrevoets et al., 1979). Direct projections of the rubrospinal tract are present to C8-T1 motoneurons that innervate distal forelimb muscles (McCurdy et al., 1987). Connections of the pyramidal tract to motoneurons are mediated by spinal interneurons. In primates both the rubrospinal and corticospinal tracts connect with C8-T1 motoneurons that innervate distal forelimb muscles (Holstege et al., 1988; Bortov and Strick, 1993). The whole system is somatotopically organized (Figure 2).

The anterior vermis and the pyramis, posterior vermal lobule VIII, also are part of the motor system. The medial vermal A zone projects to the fastigial and vestibular nuclei, the X zone to the interstitial cell groups, the B zone to the lateral vestibular nucleus. These zones supervise the long descending brain stem pathways, with the interstitial cell groups giving rise to branching axons with spinal and thalamic targets. Sugihara and Shinoda (2004) found the A zone and the adjoining A2 zone (a zone that has only been identified in the rodent cerebellum) to be composed of a number of narrow alternating zebrin-positive and negative- subzones (Figure 1E). Each of these zones receives climbing fibers carrying somatosensory, vestibular or tectal information input from particular regions of the caudal medial accessory olive (MAO). The share of each of these subzones in the corticonuclear projections to the fastigial and vestibular nuclei, and their connections with the brain stem motor systems remains to be established. Although it has been shown in mice that the ipsilateral fastigiobulbar pathway is glycinergic (Bagnall et al., 2009), we do not know which Purkinje cells provide the input to these inhibitory neurons.

## Cerebellar efferent pathways and climbing fiber paths to the C2, D1 and D2 zones are organized as closed loops

The zebrin-positive zones C2, D1 and D2 that alternate with the zebrin-negative zones in the “motor” regions of the cerebellum extend beyond these regions in the hemisphere of the ansiform lobule and the paraflocculus. These lobules, lacking zebrin-negative zones are entirely zebrin-positive and the borders between the zones cannot be determined from the zebrin pattern.

**Figure 3:**
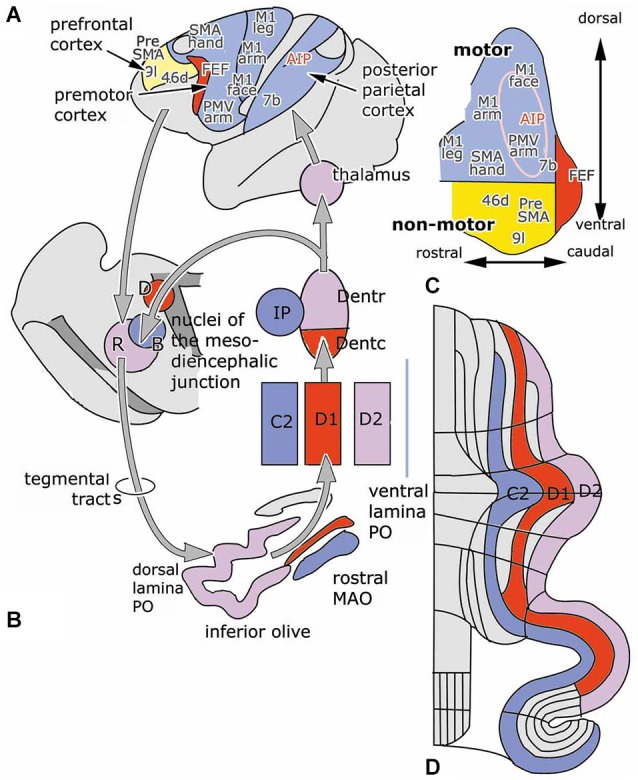
**(A)** Diagram of the left cerebral hemisphere of a monkey. Areas receiving input from the rostral motor dentate (blue), the caudal non-motor dentate (yellow) and the caudal pole of the dentate (red), as indicated in panel **(C)**, a diagram of the functional division of the monkey dentate nucleus according to Strick et al. (2009); are indicated. **(B)** Circuitry of the C2, D1 and D2 zones. **(D)** Diagram of the flattened cerebellar cortex showing the position of the C2, D1 and D2 zones. Abbreviations: AIP, anterior intraparietal area; B, Bechterew’s nucleus; D, Darkschewitsch nucleus; Dentc/r, caudal/rostral dentate nucleus; FEF, frontal eye field; IP, posterior interposed nucleus; MAO, medial accessory olive; PO, principal olive; R, parvocellular red nucleus; SMA, supplementary motor area; a, anterior; p, posterior.

With some exceptions, the cerebello-cerebral pathways were found to be crossed. Strick et al. (2009) distinguished a rostromedial “motor” area of the dentate that projects in a somatotopical manner to motor, premotor and parietal areas, and a more ventral and caudal “non-motor” part that is connected with prefrontal and parietal areas. The caudal pole of the dentate projects to the frontal eye field (Figure 3, inset). In this review, rostral and caudal portions of the dentate have been distinguished as target nuclei of the D2 and D1 zones, that are also distinguished by their climbing fiber projections from the dorsal and ventral lamina of the principal olive and their differential projection to the parvocellular red nucleus. It seems most likely, if not proved, that both Strick’s rostral, “motor” and caudal “non-motor” divisions of the monkey dentate should be included in the target nucleus of the D2 zone, and that its caudal, visual pole serves as the target nucleus of the D1 zone. In accordance with this the circuitry of the D1 zone is shown in red in Figure 3. As a consequence, the D2 zone would consist of motor and non-motor segments. The motor segments would be located in the lateral hemisphere of the motor divisions of the cerebellum. This localization is supported by the observations in transneuronal labeling experiments illustrated in Figures 5–7.

**Figure 4:**
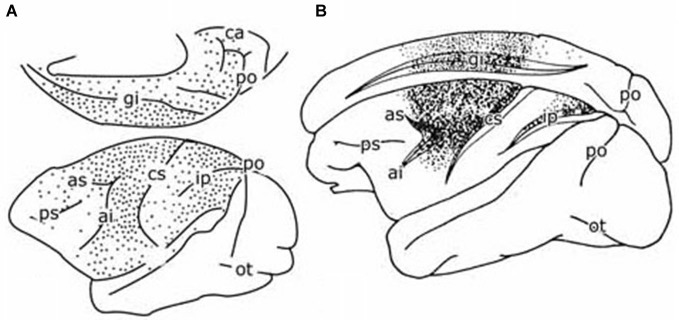
**(A)** Diagram of the cerebral hemisphere of a monkey showing the distribution of retrogradely labeled cells from a large injection of a retrograde tracer in the pontine nuclei. Redrawn from Glickstein et al. (1985). **(B)** Diagram of the cerebral hemisphere of a monkey showing the distribution of retrogradely labeled cells from an injection of a retrograde tracer in the parvocellular red nucleus of a monkey. Redrawn from Humphrey et al. (1984). Abbreviations: ai, inferior limb arcuate fissure; as, superior limb of arcuate fissure; ca, calcerine fissure; ci, central fissure; gi, cingulate fissure; Ip, intraparietal fissure; ot, occipito-temporal fisure; po, parieto-occipital fissure; ps, principal fissure.

**Figure 5:**
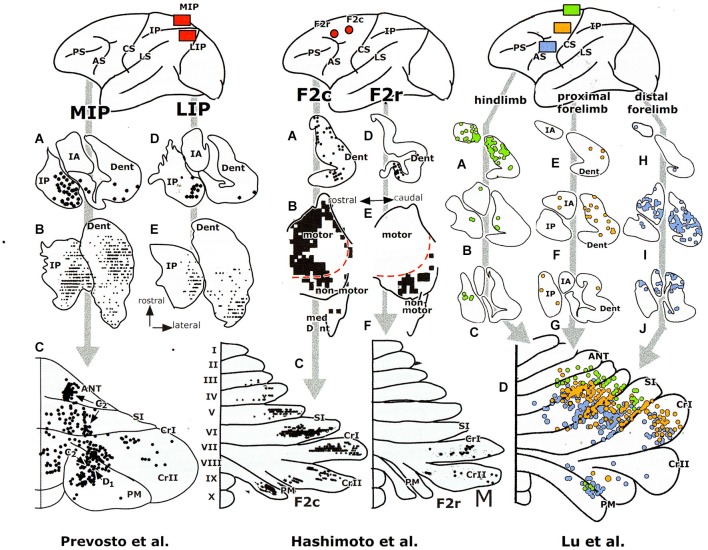
**Retrograde transneuronal labeling experiments with injections of the tracer in areas of the cerebral cortex of a monkey, showing labeling in the cerebellar nuclei (A–J) and of Purkinje cells in the diagrams of the cerebellar cortex (lower panels)**. Redrawn from Hashimoto et al. (2010) and Prevosto et al. (2010). Abbreviations: ANT, anterior lobe; AS, arcuate sulcus; CRI/II, Crus I /II; CS, central sulcus; Dent, dentate nucleus; F2c/r, caudal/rostral dorsal premotor area; IPAR, intraparietal sulcus; IP, posterior interposed nucleus; LS, lateral sulcus; IA, anterior interposed nucleus; MIP, medial intraparietal area; LIP, lateral intraparietal area; PM, paramedian lobule; PS, principal sulcus; SI, simplex lobule.

**Figure 6:**
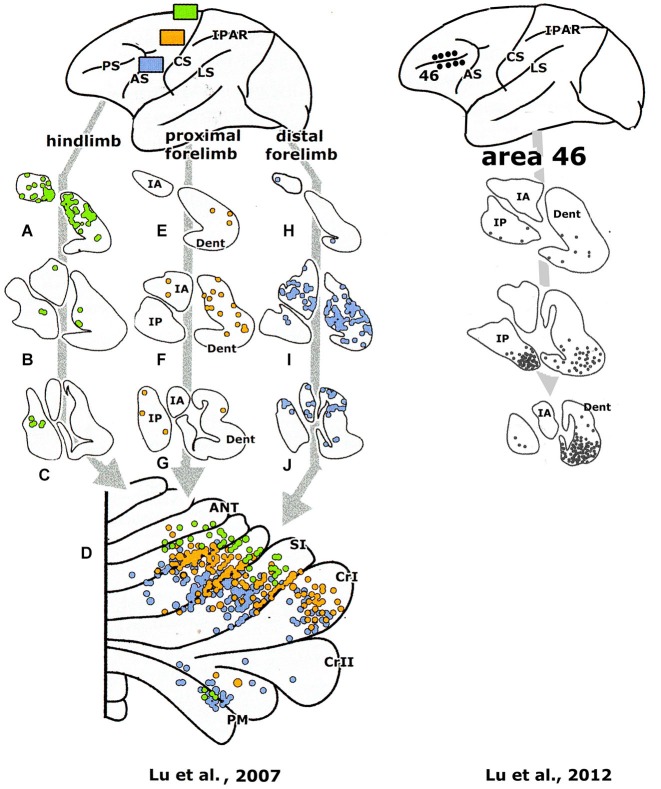
**Retrograde transneuronal labeling experiments with injections of the tracer in areas of the cerebral cortex of a monkey, showing labeling in the cerebellar nuclei (A–J) and of Purkinje cells in the diagrams of the cerebellar cortex (lower panels)**. Redrawn from Lu et al. (2007, 2012), For abbreviations see Figure 5.

**Figure 7:**
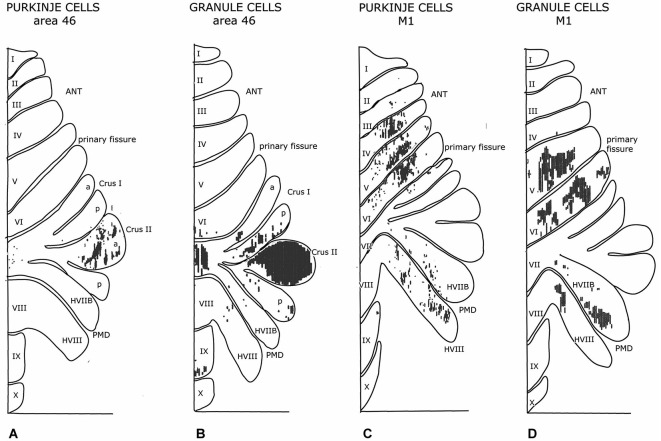
**Diagrams of the monkey cerebellum showing transneuronal retrograde labeling of Purkinje cells and transneuronal antegrade labeling of mossy fibers after injections of tracers in prefrontal area 46 and the primary motor cortex**. Note similarity in the localization of Purkinje cells and granule cells labeled from the same cortical area. Redrawn from Kelly and Strick (2003). Abbreviations: ANT, anterior lobe; PMD, pramedian lobule.

The climbing fiber circuitry of the zebrin-positive zones differs from the zebrin-negative zones (Figure 4). Their olivary subnuclei, the rostral (MAO) and the principal olive, receive their afferents from nuclei located at the meso-diencephalic border, the parvocellular red nucleus and the nuclei of Darkschewitsch and Bechterew (Onodera, 1984). Direct cortico-olivary projections appear to be few. The rostral MAO that innervates the C2 zone receives its afferents from Darkschewitsch nucleus, the ventral lamina of the principal olive that innervates D1 from Bechterew’s nucleus, corresponding to the dorsomedial portion of the parvocellular red nucleus in primates, and the dorsal lamina of the principal olive, that innervates D2, from the main lateral portion of the parvocellular red nucleus (Strominger et al., 1979). These nuclei receive a double input, both from the target nuclei of the zebrin-positive zones, the posterior interposed and dentate nuclei, and from the cerebral cortex. The posterior interposed nucleus projects to Darkschewitsch nucleus (Voogd, 1964), the rostral dentate to the main lateral and caudal portion of the parvocellular red nucleus (Flumerfelt et al., 1973) and the caudal dentate to the dorsomedial subnucleus of the parvocellular red nucleus (van Kan et al., 1993, see Voogd and Ruigrok, 2004). These zebrin-positive zones, therefore, are included in large closed cerebello-mesencephalo-olivary circuits. The central tegmental tract, the pathway from the parvocellular red nucleus to the dorsal lamina of the inferior olive belongs to the largest systems in the human brain stem.

The question, whether the cerebello-thalamocortical and corticorubro-olivary loops are also reciprocally organized remains. In monkeys the target nucleus of the D2 zone, the rostral dentate, projects to the primary motor and several premotor areas,to parietal area7b, the medial (MIP) and lateral (LIP) intraparietal areas and prefrontal areas 9l, 46d, preSMA and the rostral dorsal premotor area (Strick et al., 2009, Figure 3; Hashimoto et al., 2010; Prevosto et al., 2010, Figure 5; Lu et al., 2007, Figure 6; Kelly and Strick, 2003, Figure 7; Kievit, 1979). Targets of the D1 zone and the caudal dentate are limited to the frontal eye field (Lynch et al., 1994, Figure 3). The posterior interposed nucleus, the target nucleus of the C2 zone, projects to tne primary motor and premotor areas, the intraparietal areas MIP and LIP, prefrontal area 46 and the frontal eye fields (Strick et al., 2009; Prevosto et al., 2010, Figure 5; Lu et al., 2012, Figure 6; Kievit, 1979; Lynch et al., 1994). Widespread projections of the posterior interposed nucleus to these cortical areas were confirmed by Sultan et al. (2012) using fMRI after electrically stimulating the cerebellar nuclei and/or the superior cerebellar peduncle. Their observation of bilateral blood-oxygenation-level dependent (BOLD) responses by these authors after stimulation of the posterior interposed nucleus or the peduncle remains unexplained.

For monkeys the presence of reciprocal projections from primary and premotor areas, the frontal eye field and the posterior parietal cortex to the parvocellular red nucleus is clear from the plot of Humphrey et al. (1984) of retrogradely labeled neurons from an injection of this nucleus (Figure 4B). Somatotopically organized projections from the primary motor and premotor areas are located in the laterocaudal red nucleus and thus target the D2 zone via the dorsal lamina of the principal olive (Kuypers and Lawrence, 1967; Hartmann von Monakow et al., 1979; Strominger et al., 1979; Orioli and Strick, 1989; Tokuno et al., 1995; Burman et al., 2000). Projections from the frontal eye fields are shunted through the dorsomedial parvocellular red nucleus and the ventral lamina of the principal olive to the D1 zone (Huerta et al., 1986; Stanton, 1988; Huerta and Kaas, 1990). The location of the posterior parietal projection to the parvocellular red nucleus is not known; a prefrontal projection to the dorsomedial red nucleus only has been substantiated for area 9 by Leichnetz (1982). Cortical afferents of Darkschewitsch nucleus, the link in the C2 circuitry, are mostly the same as for the parvocellular red nucleus: motor cortex, frontal eyefields, prefrontal cortex and the posterior parietal area (Faugier-Grimaud and Ventre, 1989; Leichnetz and Gonzalo-Ruiz, 1996). Caudal, motor and rostral, visual receiving, parts of the rostral MAO were distinguished by Porter et al. (1993) in the cat. These divisions, presumably, supply climbing fibers to the anterior and posterior motor divisions, and the hemisphere of the ansiform lobule and the paraflocculus, respectively. Similar laterocaudal visuomotor and rostromedial skeletomotor divisions also have been recognized in the posterior interposed nucleus (van Kan et al., 1993).

Evidence for the reciprocity in the cerebello-cortical climbing fiber loops is still incomplete, although more complete for the monkey than for the cat; studies with modern tracing methods on these connections have not yet been published. Cortical afferents in the circuitry of the C2 and D2 zones appear to be very similar, the D2 zone and its tributaries, standing out as the largest system in primates. In cetacea the C2 zone occupies this prominent position (Korneliussen, 1968). Remarkably, the interaction of cerebellar and cortical afferents in the parvocellular red nucleus and the nucleus of Darkschewitsch, and the physiology of the recurrent climbing fiber loops has never been studied.

It should be remembered that the projections of the dentate and interposed nuclei are not limited to the thalamus and the cortex. Branching axons of the anterior interposed nucleus terminate in the contralateral magnocellular red nucleus and in the nucleus reticularis tegmenti pontis. The posterior interposed nucleus projects to parts of the reticular formation, to a medial ridge of cells along the red nucleus and to the tectum and pretectum. Apart from its projection to the parvocellular red nucleus the dentate is also connected with the pontine nuclei and the nucleus reticularis tegmenti pontis. The rostral dentate projects to the main, lateral and caudal parvocellular red nucleus, its caudal part to the dorsomedial subnucleus of the parvocellular red nucleus and to the tectum and the pretectum (Teune et al., 2000; Ruigrok and Teune, 2014).

Few studies have appeared on climbing fiber afferent systems using MRI. According to Diedrichsen et al. (2010) the BOLD signal in the cerebellum in fMRI mostly depends on the mossy fiber input and obscures the activity in the Purkinje cells and the climbing fibers. One study of the cortico-rubral relations in the human brain, using brain resting state functional connectivity, showed correlations of signal intensity of the red nucleus and insular, hippocampal, occipital and prefrontal cortices, but not with motor and premotor areas (Nioche et al., 2009). Jang et al. (2012), using diffusion tensor tractography, found direct projections to the inferior olive from the sensorimotor and posterior parietal cortex, in addition to the red nucleus and the pontine nuclei. Thus far the conclusions of these studies differ substantially from the experimental findings.

The picture that emerges of the motor regions of the cerebellum is that of an array of intercallated modules with peripheral motor and more extensive motor and non-motor cortical connections. The latter are part of a system of closed cerebello-rubral and cortical climbing fiber loops. Parallel fibers traverse Purkinje cells of these different modules and effectuate a cerebellar output serving adaptation or coordination of the motor system. These vague terms hide our lack of knowledge on what is actually happening at the brain stem and cortical targets of the modules.

## Is each Purkinje cell zone a basic functional unit?

Simpson (2011) pointed out that what is missing in the old postulate that each Purkinje cell zone constitutes a basic functional unit of the cerebellum, are tractable behaviors with which to evaluate hypothesis about the functions performed by the zones in relation to each other. This kind of behavior can be studied by stimulation or inactivation of a Purkinje cell zone, its cerebellar or vestibular target nucleus or its climbing fiber afferents.

Reaching and grasping were studied by Mason et al. (1998) in monkeys after inactivation of small parts of the cerebellar nuclei by the injection of muscimol. In accordance with the somatotopical localization in the anterior interposed and rostral dentate nuclei, discussed before, they found deficits in grasping with the hindlimb in the most anterior injections of these nuclei and with the forelimb with injections in their more caudal parts. Injections in the posterior interposed nucleus and the adjoining dentate resulted in deficits in aiming of reach and stability of the arm. These experiments suggest that the posterior interposed nucleus, with the C2 zone, and the dentate, with the D2 zone, are involved in motor functions of the cerebellum. But how? Mason et al. (1998) suggested that the medial strip of the magnocellular red nucleus, that receives a projection of the posterior interposed nucleus is involved. No such a brainstem relay is known for the rostral dentate.

Horn et al. (2010) defined functions of cerebellar modules by inactivating their olivary subnuclei in the cat. Deletion of the complex spikes causes an increase of the simple spikes, a high rate of the Purkinje cell discharge and inhibition of the output of the modules. They studied possible deficits in the reach-to-grasp where the cat grasps a handle on a tone cue to get a reward, and locomotion. Inactivation of the C1, C3 and Y modules from the rostral DAO caused contralateral deficits in gripping the handle with the paw, paw dragging during locomotion and maintaining limb position during stance. With inactivation of the C2 module from the rostral MAO there are less problems with grasping but, stance and reach trajectory and locomotion are seriously affected. These observations are very similar to those of Mason et al. (1998) on inactivation of the anterior and posterior interposed nuclei in the monkey.

Deficits similar to inactivation of the rostral DAO had been found with lesions of the anterior interposed nucleus or the contralateral red nucleus (Gibson et al., 1994). But the deficits caused by inactivation of the rostral MAO could not be reproduced by lesions of the contralateral red nucleus.

Martin et al. (2000) found under reaching in a reach to grasp task in cats after inactivation of the anterior interposed nucleus and overreaching when the posterior interposed nucleus was affected. Under- and overreaching were also found after inactivation of the projection areas of the anterior and posterior interposed nuclei in the rostromedial and rostrolateral motor cortex, respectively. However, undereaching rather than overreaching was found by Horn et al. (2010) after inactivation of the rostral MAO.

Pijpers et al. (2008) injected a neurotoxin in the hindlimb region of the C1 zone in the copula pyramidis of the rat. This caused local degeneration of mossy and climbing fibers. Climbing and mossy fiber collaterals that innervate rostral parts of the C1 and related zebrin-negative zones also are affected. Inactivation of hindlimb segments of the C1 and related zones did not affect skilled walking or the overall stepping pattern but reduced step-dependent modulation of cutaneous reflexes. Paw-dragging of the forelimb, as observed by Horn et al. (2010) in the cat could not observed in the rat because in Pijpers’ experiments the hindlimb rather than the forelimb segments of the C1 zone were injected.

Seone et al. (2005) found greater control of axial muscles in rats treated with a toxin that preserves the caudal MAO than in animals treated with another toxin that preserves the rostral MAO. Reaching and grasping were not studied by these authors. In a critical review of these studies Cerminara (2010) pointed out that it is premature to conclude, as Horn et al. (2010) did, that “… each module has a specific and unique function in sensory-motor integration”. True, restriction to a single module could not be guaranteed in any of the relevant studies. The injections of the toxin in the experiments of Horn et al. (2010) may have affected more than one subdivision of the olive, degeneration of climbing and mossy fiber collaterals with toxin injections of the caudal C1 zone in the experiments of Pijpers et al. (2008) deafferented other Purkinje cell zones in the rostral cerebellum, and the toxins used by Seone et al. (2005) preserved 47–59% of the neurons in other subdivisions of the olive apart from the rostral and caudal MAO. Still, in my opinion, involvement of the principal olive in the experiments of Horn et al. (2010) with injections of the rostral MAO or DAO is unlikely to have influenced their observations on the reach to grasp and locomotion (Milak et al., 1997; Martin et al., 2000). An extension of their rostral MAO injection into the caudal MAO was never observed by these authors. Even when other zones than the injected C1 zone in the experiments of Pijpers et al. (2008) would have been deafferented, they would belong to the same C1, C3, Y category. Other confusing factors mentioned by Cerminara (2010), such as the mutual inhibition of microzones or electrotonic coupling do not cross the spaces between the olivary subdivisions and, therefore, cannot have been involved.

In evaluating the function of Purkinje cell zones their composite nature has to be taken into account. The caudal MAO innervates at least 4 subzones both in the A zone and the A2 zone, the latter being present in rodents only. Specific regions in the C3 zone have been identified as the link in the conditioned eyeblink response (Jirenhed et al., 2007). Similar to the motor effects observed by Horn et al. (2010) eyeblink resposes of the C3 zone are relayed by the anterior interposed nucleus and the contralateral red nucleus (Morcuendo et al., 2002; Pacheco-Calderón et al., 2012). In the rat the relay is a separate population of neurons in the dorsolateral red nucleus and the adjoining pararubral area with projections to the facial motor nucleus (Ruigrok and Cella, 1995). In the cat two antagonistic groups of neurons occur in the anterior interposed nucleus. A-cells fire during the active contraction of the orbicularis oculi muscle, B-cells stop firing during downward displacement of the upper eyelid. A-cells project to the red nucleus, B-cells would project to the perioculomotor area, a projection that has not yet been verified anatomically (Sánchez-Campusano et al., 2012; Perciavalle et al., 2013). Visually dominated segments of the C2 zone do not participate in the initiation or storage of acquired memories in the conditioned eyeblink response, but play an enhancer role in the performance and the proper timing of the reflex (Jiménez-Díaz et al., 2004).

Behavioral studies of the D1 and D2 zones are available for its Crus I segments only; the anterior lobe, the paramedian lobules and the paraflocculus have not yet been studied. Purkinje cells of the Crus I D1 zone in the cat were found to encode the motion of a target, a moving tube in front of the animal, and the visual GO signal for the cat to reach for food in the tube (Miles et al., 2006). Purkinje cells in the adjoining Crus I D2 zone also encode target motion, but tonic spike activity was maintained during the transient disappearance of the target. This Purkinje cell activity may reflect the operation of an internal model based on memory of its previous motion (Cerminara et al., 2009; Cerminara and Apps, 2013). Whether such a model is also operative in the D1 zone is not known. Visual climbing fiber input has been documented for the D1 zone (Edge et al., 2003, see also section III) but not for D2. Visual mossy fiber input in the cat, relayed by the pontine nuclei, reaches the Crus I, but has not been studied in detail (Mower et al., 1980; Xiong et al., 2002).

Proville et al. (2014) found partially overlapping projections from the pontine whisker sensory and motor projections to the Crus I D2 zone in mice. Recurrent projections from these Purkinje cells excite neurons in lamina V of the whisker motor cortex and are responsible for the fine modulation of the whisker movement parameters observed in these experiments.

Transneuronal retrograde labeling in the caudal non-motor dentate and of Purkinje cells in the ansiform lobule (Hashimoto et al., 2010, Figure 5) or in the Crus II only (Kelly and Strick, 2003, Figure 7) was found with injections in the rostral dorsal premotor area (which is considered as a prefrontal area because it lacks connections with the primary motor cortex) and prefrontal area 46, respectively. This supports the notion of Crus II D2 zone being one of the main non-motor areas of the cerebellum.

Function is best-known for the Purkinje cell zones of the cerebellar flocculus that are part of the circuitry subserving compensatory eye movements in the planes of the horizontal and anterior semicircular canals (Van der Steen et al., 1994; Voogd et al., 2012). Behavioral paradigms for other Purkinje cell zones are still scarce, and the nature of the interactions of the cerebellum at brain stem motor centers or at the level of the cerebral cortex remain largely unknown.

## Topographical relations between mossy and climbing fibers in the cerebellar cortex

**Figure 8:**
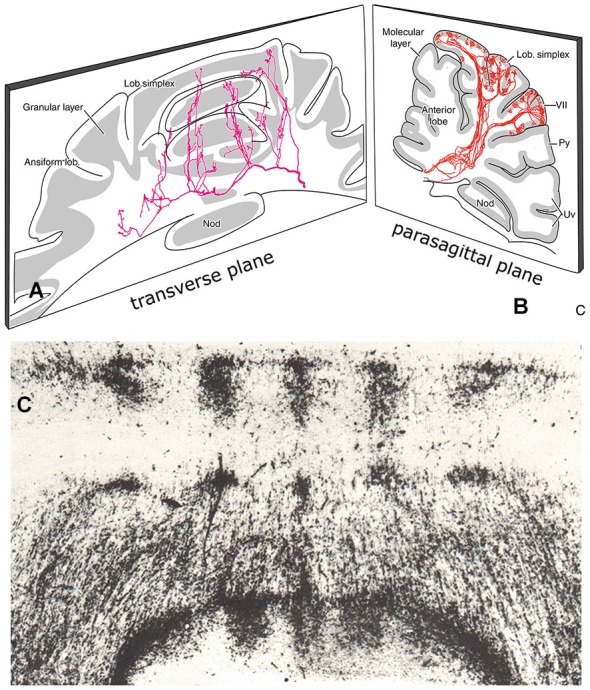
**(A)** Transverse section through the cerebellum of the rat showing course and collateralization of a reticulocerebellar mossy fiber. The collaterals terminate as longitudinal aggregates of mossy fiber terminals. Redrawn from Wu et al. (1999). **(B)** Parasagittal section showing branching olivocerebellar fibers from a small injection of an antegrade tracer in the inferior olive. The branches terminate in a narrow longitudinal strip of climbing fibers in the molecular layer, Redrawn from Sugihara et al. (1999). **(C)** Transverse section through the anterior lobe of the cerebellum of the tree shrew (Tupaia glis) showing distribution of the spinocerebellar mossy fibers. Abbreviations: Nod, nodulus; Py, pyramis; Uv, uvula.

Purkinje cell zones of the hemisphere receiving peripheral somatosensory climbing fiber input (C1, C3 and Y zones) can be subdivided into microzones. Microzones are narrow longitudinal strips of Purkinje cells that receive climbing fiber input sharing the same receptive field (Andersson and Oscarsson, 1978). Microzones were visualized by Sugihara et al. (2001) as longitudinal strips of branching olivocerebellar fibers (Figure 8B). This type of branching was observed in all parts of the cerebellum. A microzonal organization, therefore, appears to be a general property of the cerebellum.

**Figure 9:**
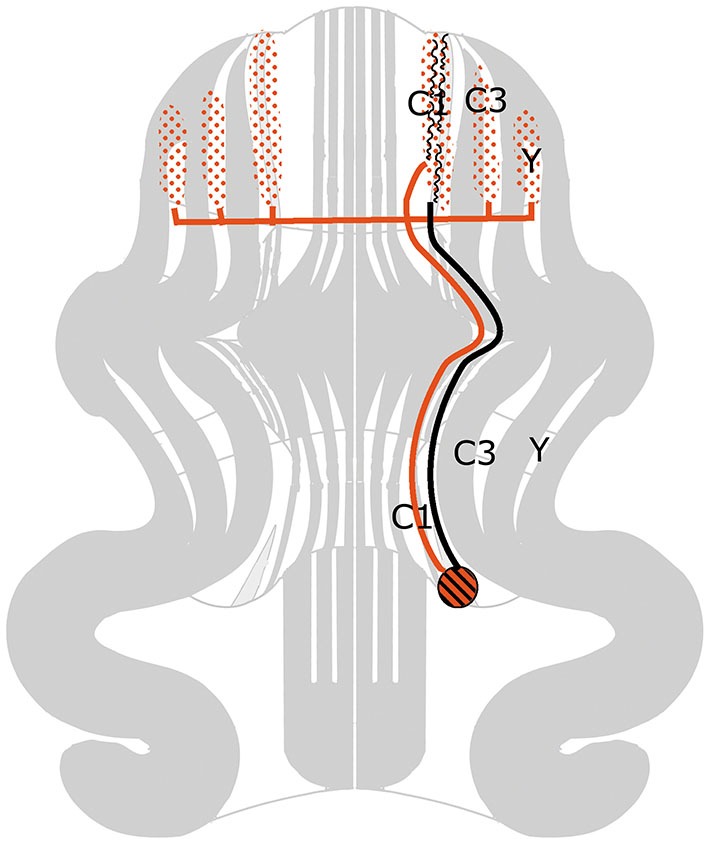
**Diagram of the flattened cerebellar cortex showing collateralization of climbing and mossy fibers from a small injection site of choleras toxin B subunit in the caudal C1 zone of the rat**. Climbing collaterals remain in the same zone. Mossy fibers collateralize subjacent to the climbing fiber collaterals and in symmetrically disposed neighboring zones of the same zebrin-signature.

Mossy fibers, initially, follow a transverse course through the cerebellar white matter (Figure 8A). At regular sites they emit thin collaterals that terminate in longitudinal aggregates of mossy fiber terminals (rosettes) in the granular layer (Scheibel, 1977; Wu et al., 1999). There is a special topographical relationship between the climbing fiber microzones and these mossy fiber terminal aggregates. Climbing and mossy fiber collateral projections were traced in the rat from small injections of cholera toxin B subunit in identified cortical zones, that included the molecular and granular layers. Climbing fiber collaterals were found to terminate in narrow strips within other segments of the same zone. Mossy fiber collaterals always terminate subjacent to the climbing fiber collaterals in the granular layer (Pijpers et al., 2006). In addition, mossy fiber collaterals terminate, bilaterally and symmetrically, as multiple longitudinal aggregates (Figures 8A, 9). Mossy fiber collaterals remain restricted to zones of the same zebrin-type of the injected zone. The convergence of mossy and climbing fiber evoked potentials from stimulation of peripheral nerves or the somatosensory cortex onto a single somatotopical map in the anterior lobe was already known from Provini et al. (1968). In the cat C3 zone, both the climbing fibers of the dorsal column spine-olivary climbing fiber path and the mossy fibers of the exteroceptive component of the cuneocerebellar mossy fiber system were found to terminate according to the same detailed somatotopical pattern (Ekerot and Larson, 1980; Garwicz et al., 1998). Similar observations for somatotopically organized climbing fiber and mossy fiber input from the basilar pontine nuclei to the copula pyramidis of the rat were made by Cerminara et al. (2013).

In the studies of Voogd et al. (2003) and Pijpers et al. (2006) the topographical relationship of climbing fiber and mossy fiber microzones was found in all parts of the cerebellum. In regions without somatotopical input we do not know the nature of this relationship. Do mossy and climbing fibers share input from the same cortical regions? Or from similar functional networks? Or from the same axis in the vestibular coordinate system (Simpson and Graf, 1985)? We do not know.

Different ideas have been proposed for the functional relations of mossy fiber terminal aggregates and the climbing fiber microzones. Llinás (1982) hypothesized that ascending segments of granule cell axons preferentially terminate on the superjacent Purkinje cell dendrites. This idea received support from Brown and Bower (2001), who demonstrated that the receptive field for a Purkinje cell complex spike is similar to the receptive field of the granule cells immediately subjacent to that Purkinje cell. Ekerot and Jörntell (2003) confirmed the similarity in receptive field organization of climbing fiber microzones and the subjacent mossy fiber terminal aggregates. However, determining the receptive field organization of parallel fibers innervating the Purkinje cells and interneurons in these microzones, they found the receptive fields of interneurons to correspond to the mossy fiber terminal aggregate of the microzone, whereas the receptive fields of Purkinje cells corresponded to mossy fibers in neighboring microzones. Llinás (1982) hypothesis, therefore, may apply to interneurons but not to Purkinje cells.The properties of the parallel fibers cannot be explained without involving learning mechanisms. Barmack and Yakhanitsa (2011) found the optimal planes for complex and simple spikes of Purkinje cells in climbing fiber zones of the uvula-nodulus innervated by the vertical semicircular canals to be identical, but oppositely polarized. This observation is not in accordance with the Llinás (1982) hypothesis.They proposed that the opposite polarization is due to inhibition of Purkinje cells by stellate cells. Stellate cells are modulated in phase with the complex spikes and out of phase with the simple spikes. Stellate cells can be excited by glutamate spillover from the discharge of adjacent climbing fibers. Golgi cells are modulated in phase with the simple spikes and, therefore, cannot be account for the modulation of simple spikes by complex spikes. The relations between mossy fiver aggregates and climbing fiber microzones, therefore, are complicated and involve interneurons and plastic changes in the circuitry.

## Similarities in the anatomical organization of mossy and climbing fiber systems

Gibson et al. (2004) and others emphasized the different properties of. mossy and climbing fibers. Climbing fibers have a strong influence on a small number of Purkinje cells, discharge at a low rate and signal externally imposed disturbances to particular regions of the body when the animal is not actively moving. Mossy fibers exert a relatively small effect on a large number of Purkinje cells via the parallel fibers; they discharge at high frequencies and carry detailed information about the external world as well as intended and actual body movement. Still, the topographical relationship of climbing fiber microzones and mossy fiber aggregates suggests similarities in the anatomical organization of both systems.

**Figure 10:**
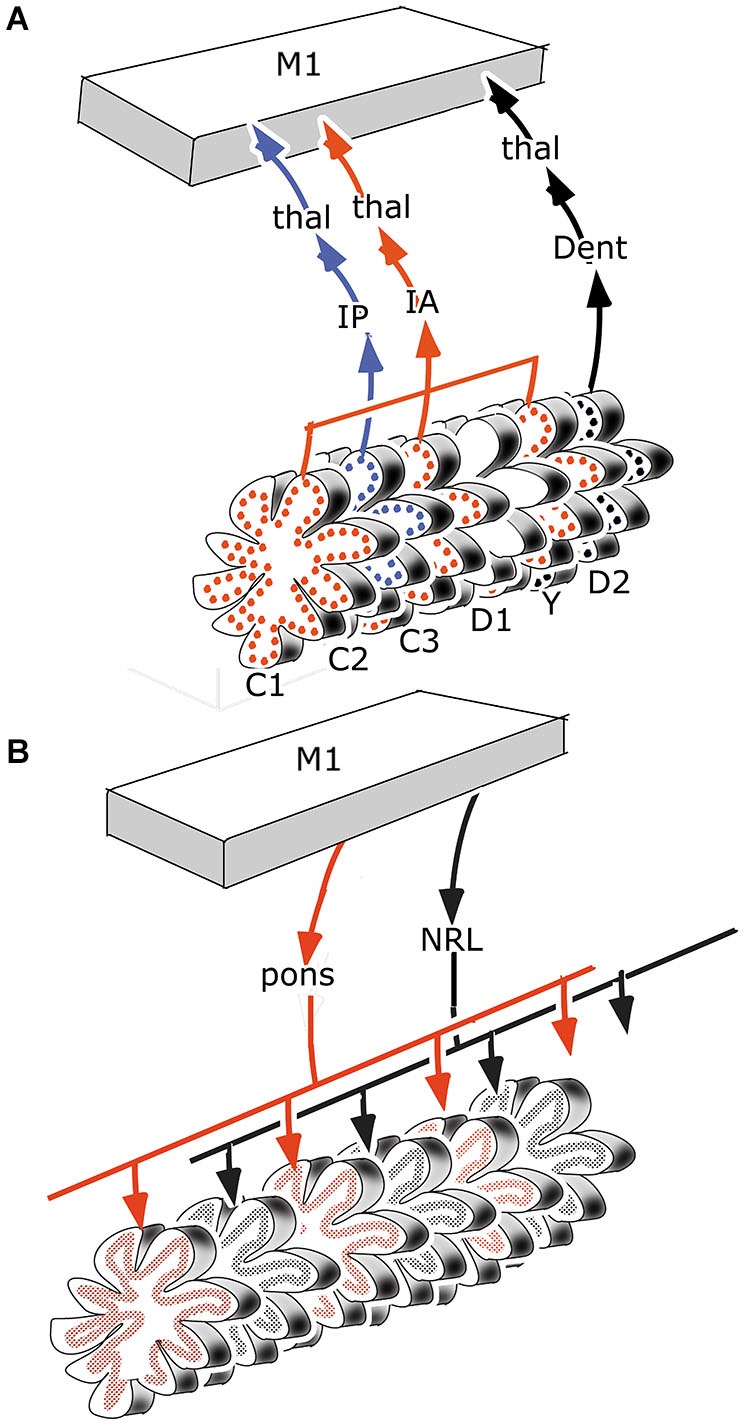
**(A)** Retrograde transneuronal retrograde labeling of Purkinje cells from the primary motor cortex would affect all modules with thalamocortical to this area, in the case of motor regions of the cerebellum they would include the C1, C2, C3, Y and D2 modules. **(B)** Antegrade transneuronal labeling from the primary motor cortex of mossy fiber terminals in the granular layer would label all mossy fiber systems with intermediate stations that receive cortical input, such as the pontine nuclei and the lateral reticular nucleus. Each of these systems would distribute in multiple parallel strips of labeled mossy fiber rosettes. Abbreviations: Dent, dentate nucleus; IA, anterior interposed nucleus; IP, posterior interposed nucleus; NRL, lateral reticular nucleus; thal, thalamus.

It has been suggested that the efferent thalamocortical and the cortico-ponto-cerebellar mossy fiber systems are organized as closed loops (Kelly and Strick, 2003, Figure 7), similar to the reciprocal circuitry of the climbing fiber system discussed in section III. Indeed, at the origin of these loops, as the cortico-rubral and corticopontine projections (Figure 4) and at their end, as the common somatotopical map in the anterior lobe, the two systems are very similar. Mossy fiber loops differ from climbing fiber loops because the latter are organized as a separate loop for each module Figure 10A), whereas mossy fiber loops always include multiple modules (Figure 10B). Moreover, the relays in recurrent mossy fiber loops are different from the climbing fiber loops. The projection of the anterior interposed nucleus to the magnocellular red nucleus is reciprocated by a collateral projection of the rubrospinal tract to this nucleus (Huisman et al., 1983). The circuitry through the nucleus reticularis tegmenti pontis and through the red nucleus and the lateral reticular nucleus was emphasized by Allen and Tsukahara (1974). Whether these connections are reciprocal at the level of the cerebellum is not known. Moreover, these systems are complicated by the presence of cortical input at the level of the reticular nucleus of the pons (Brodal and Brodal, 1971) and by the termination of the ipsilateral forelimb tract in the lateral reticular nucleus (Clendelin et al., 1974). The electrophysiology of these convergent inputs has not been studied.

Cerebellar-cortical connections and the cortico-cerebellar mossy fiber projections in monkeys have been found to be reciprocally organized (Kelly and Strick, 2003). Injections of a retrograde transneuronal tracer in the forelimb motor cortex labeled Purkinje cells in the anterior lobe hemisphere and in the paramedian lobule. Granule cells labeled from an injection of an antegrade transneuronal tracer were found in the same lobules. Injections in prefrontal area 46 labeled both types of neurons in the Crus II (Figure 7). Injections of the primary motor cortex would be expected to label Purkinje cells in the C1, C2, C3 and D2 zones, and injections of area 46 in the C2 and D2 zones, all of which project to these cortical area through their target nuclei (section III). Transneuronal labeling of granule cells from injection sites in the same areas would label mossy fibers from all precerebellar nuclei that receive cortical input: apart from the pontine nuclei these would include reticular, dorsal column and even spinocerebellar nuclei. Moreover retrogradely labeled mossy fibers would distribute widely, collateralizing to different Purkinje cell zones. True reciprocity, therefore, has not been demonstrated in this study.

## Topographical relations of different mossy fiber system in the cerebellum

Where individual mossy fibers emit collaterals bilaterally at specific medio-lateral positions, entire mossy fiber systems all terminate in bilaterally distributed discrete aggregates of mossy fiber rosettes (Figure 8C). This parcellated distribution has been found for all mossy fiber systems, spinocerebellar, cuneocerebellar (Voogd, 1969), trigeminocerebellar (Ikeda and Matsushita, 1992), reticulocerebellar (Wu et al., 1999), vestibulocerebellar (Matsushita and Wang, 1987) and pontocerebellar systems (Serapide et al., 2001). Antegrade axonal tracing studies of different mossy fiber systems have been published for cats and rats, but scarcely any are available for primates. The last study on the spinocerebellar tracts in monkeys dates from the 19th century. Little is known of the interrelations of different mossy fiber systems in the cortex. The relation of mosssy fiber terminal fields to the zebrin-positive and -negative Purkinje cell zones was studied for the spino- and cuneocerebellar mossy fiber projection in the anterior vermis of the rat (Ji and Hawkes, 1994). In this case the two systems were found to interdigitate, an observation confirmed by Gebre et al. (2012). Similar information on other mossy fiber system is not available.

**Figure 11:**
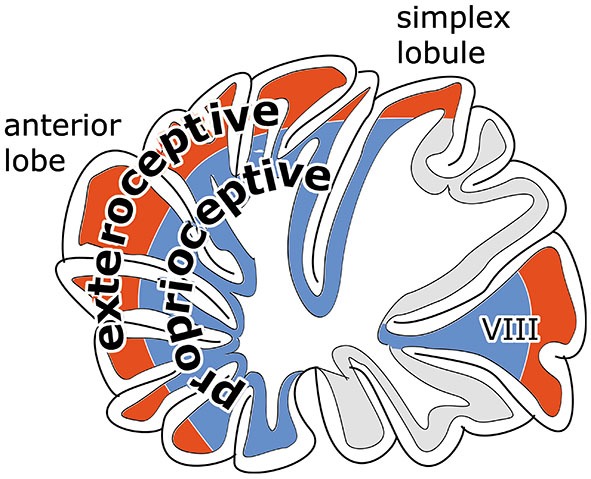
**Diagram illustrating concentric arrangement of exteroceptive components of mossy fiber systems and pontocerebellar mossy fibers to superficial and of proprioceptive and vestibular mossy fibers to the cortex in the bottom of the fissures**.

Another feature of the distribution of mossy fibers in motor regions of the cerebellum is their concentric arrangement (Figure 11). Corticopontine and exteroceptive components of mossy fiber systems terminate in apical parts of the lobules, proprioceptive components and vestibular mossy fibers in the bottom of the fissures (Ekerot and Larson, 1972; Voogd and Ruigrok, 2004). This configuration raises an interesting question on the convergence of Purkinje cell axons onto the cerebellar nuclei. Do superficial and deep Purkinje cells of a lobule converge upon the same nuclear neurons? In this case lobules would have an integrating function,apart from increasing the surface area of the cerebellar cortex.

Length and synaptic size of parallel fibers differ for the upper and lower molecular layer (see Van der Want et al. (1985) for references). A relation with differences in mossy fiber input, of spinocerebellar mossy fibers to deep and pontocerebellar mossy fibers to more superficial parts of the granular layer has been suggested (Eccles et al., 1967) but has not been studied since.

## Conclusions

Repairing gaps in our knowledge on cerebellar systems asks for a collaborative effort of anatomists and physiologists. Noninvasive techniques for the tracing of axonal pathways will have to be developed to collect information in non-human primates. MRI technology will have to be improved to visualize climbing fiber paths and Purkinje cell activity. The alternation of Purkinje cell zones receiving peripheral and cortical climbing fiber input, and the contribution of the mutiple narrow Purkinje cell zones to cerebellar function should be evaluated in suitable animal models. But even when we know the precise interrelations of different cortical areas and brainstem centers with the cerebellum, the contribution of the cerebellum to the information processing in these structures remains incompletely known.

## Conflict of interest statement

The author declares that the research was conducted in the absence of any commercial or financial relationships that could be construed as a potential conflict of interest.

## References

[B1] Allen G. Tsukahara N. (1974). Cerebrcerebellar communication systems. Physiol. Rev. 54, 957–1006. 4370744 10.1152/physrev.1974.54.4.957

[B2] Andersson G. (1984). Demonstration of a cuneate relay in a cortico-olivo-cerebellar pathway in the cat. Neurosci. Lett. 46, 47–52. 10.1016/0304-3940(84)90197-6 6728328

[B3] Andersson G. Nyquist J. (1983). Origin and sagittal termination areas of cerebro-cerebellar climbing fibre paths. J. Physiol. 337, 257–285. 6875930 10.1113/jphysiol.1983.sp014623 PMC1199106

[B4] Andersson G. Oscarsson O. (1978). Climbing fiber microzones in the cerebellar vermis and their projection to different groups of cells in the lateral vestibular nucleus. Exp. Brain Res. 32, 565–579. 10.1007/bf00239553 689129

[B5] Apps R. Garwicz M. (2000). Precise matching of olivo-cortical divergence and cortico-nuclear convergence between somatotopically corresponding areas in the medial C1 and medial C3 zones of the paravermal cerebellum. Eur. J. Neurosci. 12, 205–214. 10.1046/j.1460-9568.2000.00897.x 10651875

[B6] Apps R. Hawkes R. (2009). Cerebellar cortical organization: a one map hypothesis. Nat. Rev. Neurosci. 10, 670–681. 10.1038/nrn2698 19693030

[B7] Bagnall M. W. Zingg B. Sakatos A. Moghadam S. H. Zeilhofer H. U. du Lac S. (2009). Glycinergic projection neurons of the cerebellum. J. Neurosci. 29, 10104–10110. 10.1523/JNEUROSCI.2087-09.2009 19675244 PMC3196611

[B8] Barmack N. H. Yakhanitsa V. (2011). Topsy Turvy: functions of climbing and mossy fibers in the vestibulo-cerebellum. Neuroscientist 17, 221–236. 10.1177/1073858410380251 21362689 PMC3148450

[B9] Bortov G. A. Strick P. L. (1993). Corticospinal terminations in two new-world primates: further evidence that corticomotoneuronal connections provide part of the neural substrate for manual dexterity. J. Neurosci. 13, 5105–5118. 7504721 10.1523/JNEUROSCI.13-12-05105.1993 PMC6576412

[B105] Brodal A. Brodal P. (1971). The organization of the nucleus reticularis tegmenti pontis in the cat in the light of experimental anatomical studies of its cerebral cortical afferents. Exp. Brain Res. 13, 90–110. 10.1007/bf00236432 5571061

[B10] Brown J. E. Bower J. M. (2001). Congruence of mossy fiber and climbing fiber tactile projections in the lateral hemisphere of the rat cerebellum. J. Comp. Neurol. 429, 59–70. 10.1002/1096-9861(20000101)429:1<59::AID-CNE5>3.0.CO;2-3 11086289

[B11] Burman K. Darian-Smith C. Darian-Smith I. (2000). Macaque red nucleus: origins of spinal and olivary projections and terminations of cortical inputs. J. Comp. Neurol. 423, 179–196. 10.1002/1096-9861(20000724)423:2<179::aid-cne1>3.0.co;2-# 10867653

[B12] Catsman-Berrevoets C. E. Kuypers H. H. Lemon R. N. (1979). Cells of origin of the frontal projection to magnocellular and parvocellular red nucleus and superior colliculus in cynomolgus monkey. An HRP study. Neurosci. Lett. 12, 41–46. 10.1016/0304-3940(79)91477-0 111171

[B13] Cerminara N. L. (2010). Cerebellar modules: individual or composite entities?. J. Neurosci. 30, 16065–16067 10.1523/jneurosci.4823-10.2010 21123553 PMC6634852

[B14] Cerminara N. L. Aoki H. Loft M. Sugihara I. Apps R. (2013). Structural basis of cerebellar micricircuits in the rat. J. Neurosci. 33, 16427–16442. 10.1523/JNEUROSCI.0861-13.2013 24133249 PMC3797368

[B15] Cerminara N. L. Apps R. (2013). Behavioural significance of cerebellar modules. Cerebellum 10, 484–494. 10.1007/s12311-010-0209-2 20838949 PMC3169775

[B16] Cerminara N. L. Apps R. Marple-Horvat D. E. (2009). An internal model of a moving visual target in the lateral cerebellum. J. Physiol. 587, 429–442. 10.1113/jphysiol.2008.163337 19047203 PMC2670054

[B17] Clendelin M. Ekerot C. F. Oscarsson O. (1974). The laterql reticular nucleusin the cat. III. Organization of component activated from ipsilateral forelimb tract. Exp. Brain Res. 21, 501–513. 10.1007/bf00237168 4442499

[B18] Diedrichsen J. Verstynen T. Schhlerf J. Wiestler T. (2010). Advances in functional imaging of the human cerebellum. Curr. Opin. Neurol. 23, 382–387. 10.1097/WCO.0B013e32833be837 20581682

[B19] Eccles J. C. Ito M. Szentagothaii J. (1967). The Cerebellum as a Neuronal Machine. Heidelberg, New York: Springer Verlag.

[B20] Edge A. L. Marple-Horvat E. Apps R. (2003). Lateral cerebellum: functional localization within crus I and correspondance to cortical zones. Eur. J. Neurosci. 18, 1468–1485. 10.1046/j.1460-9568.2003.02873.x 14511327

[B21] Ekerot C. F. Jörntell H. (2003). Parallel fiber receptive fields: a key to understanding cerebellar operation and learning. Cerebellum 2, 101–109. 10.1080/14734220309411 12880177

[B22] Ekerot C. F. Larson B. (1972). Differential termination of the exteroceptive and proprioceptive components of the cuneocerebellar tract. Brain Res. 36, 420–424. 10.1016/0006-8993(72)90748-2 5009648

[B23] Ekerot C. F. Larson B. (1980). Termination in overlapping sagittal zones in cerebellar anterior lobe of mossy and climbing fiber paths activated from dorsal funiculus. Exp. Brain Res. 38, 163–172. 10.1007/bf00236737 7358102

[B24] Faugier-Grimaud S. Ventre J. (1989). Anatomic connections of inferior parietal cortex (area 7) with subcortical structures related to vestibulo-ocular function in a monkey (Macaca fascicularis). J. Comp. Neurol. 280, 1–14. 10.1002/cne.902800102 2465325

[B25] Flumerfelt B. A. Otabe S. Courville J. (1973). Distinct projections to the red nucleus from the dentate and interposed nuclei in the monkey. Brain Res. 50, 408–414. 10.1016/0006-8993(73)90742-7 4196194

[B26] Garwicz M. Jörntell H. Ekerot C.-F. (1998). Cutaneous receptive fields and topography of mossy fibres and climbing fibres projecting to cat cerebellar C3 zone. J. Physiol. 512, 277–293. 10.1111/j.1469-7793.1998.277bf.x 9729638 PMC2231180

[B27] Gebre S. A. Reeber S. L. Sillitoe R. V. (2012). Parasagittal compartmentation of cerebellar mossy fibers as revealed by the patterned expression of vesicular glutamate transporters VGLUT1 and VGLUT2. Brain Struct. Funct. 217, 165–180. 10.1007/s00429-011-0339-4 21814870

[B29] Gibson A. R. Horn K. M. Pong M. (2004). Activation of climbing fibers. Cerebellum 3, 212–221. 10.1080/14734220410018995 15686099

[B30] Gibson A. R. Horn K. M. van Kan P. M. (1994). “Grasping cerebellar function,” in Insights in the Reach to Grasp, eds Hennett K. M. B. Castiello U. (Amsterdam: Elsevier), 85–108.

[B31] Gibson A. R. Robinson A. R. Alam J. Houk J. C. (1987). Somatotopic alignment between climbing fiber input and nuclear output of the cat intermediate cerebellum. J. Comp. Neurol. 260, 362–377. 10.1002/cne.902600304 3597837

[B106] Glickstein M. May J. G. Mercier B. E. (1985). Corticopontine projection in the macaque: the distribution of labeled cortical cells after large injections of horseradish peroxidase in the pontine nuclei. J. Comp. Neurol. 15, 235–259. 10.1002/cne.902350306 3998215

[B32] Hartmann von Monakow K. Akert K. Künzle H. (1979). Projections of precentral and premotor cortex to the red nucleus and other midbrain areas in Macaca fascicularis. Exp. Brain Res. 34, 91–105. 10.1007/bf00238343 83242

[B33] Hashimoto M. Takahara D. Hirata Y. Inoue K. Miyachi S. Nambu A.. (2010). Motor and non-motor projections from the cerebellum to rostrocaudally distinct sectors of the dorsal premotor cortex in macaques. Eur. J. Neurosci. 31, 1402–1413. 10.1111/j.1460-9568.2010.07151.x 20384784

[B34] Hawkes R. Leclerc N. (1987). Antigenic map of the rat cerebellar cortex: the distribution of parasagittal bands as revealed by monoclonal anti-Purkinje cell antibody mapQ113. J. Comp. Neurol. 256, 29–41. 10.1002/cne.902560104 3546410

[B107] Holstege G. Blok B. F. Ralston D. D. (1988). Anatomical evidence for red nucleus projections to motoneuronal cell groups in the spinal cord of the monkey. Neurosci. Lett. 95, 97–101. 10.1016/0304-3940(88)90639-8 2465513

[B35] Horn K. M. Pong M. Gibson A. R. (2010). Functional relations of cerebellar modules of the cat. J. Neurosci. 30, 9411–9423. 10.1523/JNEUROSCI.0440-10.2010 20631170 PMC3865504

[B36] Huerta M. F. Kaas J. H. (1990). Supplementary eye field as defined by intracortical microstimulation: connections in macaques. J. Comp. Neurol. 293, 299–330. 10.1002/cne.902930211 19189718

[B37] Huerta M. F. Krubitzer L. A. Kaas J. H. (1986). Frontal eye field as defined by intracortical microstimulation in squirrel monkeys, owl monkeys and macaque monkeys: I. Subcortical connections. J. Comp. Neurol. 253, 415–439. 10.1002/cne.902530402 3793998

[B38] Huisman A. M. Kuypers H. G. Condé F. Keizer K. (1983). Collateral od rubrospinal neurons to the cerebellum in rat. A retrograde fluorescent double labelling study. Brain Res. 264, 181–196. 10.1016/0006-8993(83)90816-8 6303500

[B39] Humphrey D. R. Gold R. Reed D. J. (1984). Sizes, laminar and topographical origins of cortical projections to the major divisions of the red nucleus in the monkey. J. Comp. Neurol. 225, 75–94. 10.1002/cne.902250109 6725640

[B108] Ikeda M. Matsushita M. (1992). Trigeminocerebellar projections to the posterior lobe in the cat as studied by antegrade transport of wheat germ agglutinin-horseradish peroxidase. J. Comp. Neurol. 316, 221–237. 10.1002/cne.903160207 1374086

[B40] Jang S. H. Chang P. H. Kwon H. G. (2012). The neural connectivity of the inferior olivary nucleus in the human brain: diffusion tensor tractography study. Neurosci. Lett. 523, 67–70. 10.1016/j.neulet.2012.06.043 22743659

[B41] Ji Z. Hawkes R. (1994). Topography of Purkinje cell compartments and mossy fiber terminal fields in lobules II and III of the rat cerebellar cortex: spinocerebellar and cuneocerebellar projections. Neuroscience 61, 935–954. 10.1016/0306-4522(94)90414-6 7530818

[B42] Jiménez-Díaz L. Navarro-López Jde D. Gruart A. Delgado-García J. M. (2004). Role of cerebellar interpositus nucleus in the genesis and control of reflex and conditioned eyelid responses. J. Neurosci. 24, 9138–9145. 10.1523/jneurosci.2025-04.2004 15483132 PMC6730068

[B43] Jirenhed D. A. Bengtsson F. Hesslow G. (2007). Acquisition, extinction and reacquisition of a cerebellar cortical memory trace. J. Neurosci. 27, 2493–2502. 10.1523/jneurosci.4202-06.2007 17344387 PMC6672498

[B44] Jörntell H. Ekerot C. G. (1999). Topographical organization of projections to cat motor cortex from nucleus interpositus anterior and forelimb skin. J. Physiol. 514, 551–566. 10.1111/j.1469-7793.1999.551ae.x 9852335 PMC2269074

[B45] Kelly R. M. Strick P. L. (2003). Cerebellar loops with motor cortex and prefrontal cortex of a nonhuman primate. J. Neurosci. 23, 8432–8444. 12968006 10.1523/JNEUROSCI.23-23-08432.2003 PMC6740694

[B46] Kievit J. (1979). Cerebello-Thalamische Projecties en de Efferente Verbindingen naar de Frontaalschors in de Rhesus Aap. Thesis. Rotterdam: Bronder.

[B47] Korneliussen H. K. (1968). Comments on the cerebellum and its division. Brain Res. 8, 229–236. 10.1016/0006-8993(68)90044-9 4871014

[B48] Kuypers H. G. Lawrence D. G. (1967). Cortical projections to the red nucleus and the brain stem in the Rhesus monkey. Brain Res. 4, 151–188. 10.1016/0006-8993(67)90004-2 4961812

[B49] Leichnetz G. R. (1982). The medial accessory nucleus of bechterew: a cell group within the anatomical limits of the rostral oculomotor complex receives a direct prefrontal projection in the monkey. J. Comp. Neurol. 210, 147–151. 10.1002/cne.902100205 7130475

[B50] Leichnetz G. R. Gonzalo-Ruiz A. (1996). Prearcuate cortex in the Cebus monkey has cortical and subcortical connections like the macaque frontal eye field and projects to fastigial-recipient oculomotor-related brainstem nuclei. Brain Res. Bull. 41, 1–29. 10.1016/s0361-9230(96)00154-2 8883912

[B51] Llinás R. (1982). General discussion: radial connectivity in the cerebellar cortex: a novel view regarding the functional organization of the molecular layer. Exp. Brain Res. Suppl. 6, 189–192 10.1007/978-3-642-68560-6_10

[B52] Lu X. Miyachi S. Ito Y. Nambu A. Takada M. (2007). Topopgraphical distribution of output neurons in cerebellar nuclei and cortex to somatotopic map of primary motor cortex. Eur. J. Neurosci. 25, 2374–2382. 10.1111/j.1460-9568.2007.05482.x 17445235

[B53] Lu X. Miyachi S. Takada M. (2012). Anatomical evidence for the involvement of medial cerebellar output from the interpositus nuclei in cognitive functions. Proc. Natl. Acad. Sci. U S A 109, 18980–18984. 10.1073/pnas.1211168109 23112179 PMC3503233

[B54] Lynch J. C. Hoover J. E. Strick P. L. (1994). Input to the primate frontal eye field from the substantia nigra, superior colliculus and dentate nucleus demonstrated by transneuronal transport. Exp. Brain Res. 100, 181–186. 10.1007/bf00227293 7813649

[B55] Martin J. H. Cooper S. E. Hacking A. Ghez C. (2000). Differential effects of deep cerebellar nuclei inactivation on reaching and adaptive control. J. Neurophysiol. 83, 1886–1899. 10758100 10.1152/jn.2000.83.4.1886

[B56] Mason C. R. Miller L. E. Baker J. F. Houk J. C. (1998). Organization of reaching and grasping movements in the primate cerebellar nuclei as revealed by focal muscimol inactivations. J. Neurophysiol. 79, 537–554. 9463420 10.1152/jn.1998.79.2.537

[B57] Matsushita M. Wang C. L. (1987). Projection pattern of vestibulocerebllar fibers in the anterior vermis of the cat: an antegrade wheat germ agglutinin-horseradish peroxidase study. Neurosci. Lett. 74, 25–30. 10.1016/0304-3940(87)90045-0 2436104

[B58] McCurdy M. L. Hansma D. I. Houk J. C. Gibson A. R. (1987). Selective projections from the cat red nucleus to digit motor neurons. J. Comp. Neurol. 265, 367–379. 10.1002/cne.902650306 2447133

[B59] Milak M. S. Shimansky Y. Bracha V. Bloedel J. R. (1997). Effects of inactivating individual cerebellar nuclei on the performance and retention of an operantly conditioned forelimb movement. J. Neurophysiol. 78, 939–959. 9307126 10.1152/jn.1997.78.2.939

[B60] Miles Ö. B. Cerminara N. L. Marple-Horvat D. E. (2006). Purkinje cells in the lateral cerebellum of the cat encode visual events and target motion during visually guided reaching. J. Physiol. 571, 619–637. 10.1113/jphysiol.2005.099382 16423861 PMC1805797

[B61] Morcuendo S. Delgado-Garcia J.-M. Ugolini G. (2002). Neuronal premotor networks involved in eyelid responses: retrograde transneuronal tracing with rabies virus from the orbicularis oculi muscle in the rat. J. Neurosci. 22, 8808–8818. 12388587 10.1523/JNEUROSCI.22-20-08808.2002 PMC6757699

[B62] Mower G. Gibson A. Robinson F. Stein J. Glickstein M. (1980). Visual pontocerebellar projections in the cat. J. Neurophysiol. 43, 355–366. 7381525 10.1152/jn.1980.43.2.355

[B63] Namba K. Sugihara I. Hahimoto M. (2011). Close correlation between the birth date of Purkinje cells and the longitudinal compartmentalization of the mouse adult cerebellum. J. Comp. Neurol. 519, 2594–2614. 10.1002/cne.22640 21456012

[B64] Nioche C. Cabanis E. A. Habas C. (2009). Functional connectivity of the human red nucleus in brain resting state at 3T. AJNR Am. J. Neuroradiol. 30, 396–403. 10.3174/ajnr.A1375 19022864 PMC7051406

[B65] Onodera S. (1984). Olivary projections from the mesodiencephalic structures in the cat studied by means of axonal transport of horseradish peroxidase and tritiated amino acids. J. Comp. Neurol. 227, 37–49. 10.1002/cne.902270106 6470209

[B66] Orioli P. J. Strick P. L. (1989). Cerebellar connections with the motor cortex and the arcuate premotor area: an analysis employing retrograde transneuronal transport of WGA-HRP. J. Comp. Neurol. 288, 612–626. 10.1002/cne.902880408 2478593

[B109] Pacheco-Calderón R. Carretero-Guillén A. Delgado-Garcia J. M. Gruart A. (2012). Red nucleus neurons actively contribute to the acquisition of classically conditioned eyelid responses in rabbits. J. Neurosci. 32, 12129–12143. 10.1523/JNEUROSCI.1782-12.2012 22933796 PMC6621528

[B67] Perciavalle V. Apps R. Bracha V. Delgado-Garcia J. M. Gibson A. R. Leggio M.. (2013). Consensus paper; current views on the role of cerebellar interpositus nucleus in movement control and emotion. Cerebellum 12, 738–757. 10.1007/s12311-013-0464-0 23564049

[B68] Pijpers A. Apps R. Pardoe J. Voogd J. Ruigrok T. J. (2006). Precise spatial relationships between mossy fibers and climbing fibers in rat cerebellar cortical zones. J. Neurosci. 26, 12057–12080. 10.1523/jneurosci.2905-06.2006 17108180 PMC6674858

[B69] Pijpers A. Winkelman B. H. Bronsing R. Ruigrok T. J. (2008). Selective impairment of the cerebellar C1 module involved in rat hind limb control reduces step-dependent modulation of cutaneous reflexes. J. Neurosci. 28, 2179–2189. 10.1523/JNEUROSCI.4668-07.2008 18305251 PMC6671855

[B70] Porter C. M. van Kan P. L. E. Horn K. M. Bloedel J. R. Gibson A. R. (1993). Functional divisions of cat rMAO. Soc. Neurosci. Abstr. 499, 10.

[B71] Prevosto V. Graf W. Ugolini G. (2010). Cerebellar inputs to intraparietal cortex areas MIP and LIP: functional frameworks for adaptive control of eye movements, reaching and arm/eye/head movement coordination. Cereb. Cortex 20, 214–228. 10.1093/cercor/bhp091 19465740 PMC2860711

[B72] Proville R. D. Spolidoro M. Guyon N. Duqué G. P. Selimi F. Isope P.. (2014). Cerebellum involvement in cortical sensorimotor circuits for the control of voluntary movements. Nat. Neurosci. 17, 1233–1239. 10.1038/nn.3773 25064850

[B73] Provini L. Redman S. Strata P. (1968). Mossy and climbing fibre organization on the anterior lobe of the cerebellum activated by forelimb and hindlimb reas of the sensorimotor cortex. Exp. Brain Res. 6, 216–233. 10.1007/bf00235125 5712701

[B74] Ruigrok T. J. Cella F. (1995). “Precerebellar nuclei and red nucleus,” in The Rat Nervous System, ed Paxnos G. (San Diego: Academic Press), 277–308.

[B76] Ruigrok T. J. Teune T. M. (2014). Collateralization of cerebellar output to functionally distinct brainstem areas. A retrograde, non-fluorescent tracing study in the rat. Front. Syst. Neurosci. 8:23. 10.3389/fnsys.2014.00023 24600356 PMC3930852

[B77] Saint-Cyr J. A. (1987). Anatomical oraganization of cortico-mesencephalo-olivary pathways in the cat as demonstratted by axonal transport techniques. J. Comp. Neurol. 257, 39–59. 10.1002/cne.902570105 2437162

[B78] Sánchez-Campusano R. Gruart A. Fernández-Mas R. Delgado-García J. M. (2012). An agonist-antagonist cerebellar nuclear system controlling eyelid kinematics during motor learning. Front. Neuroanat. 6:8. 10.3389/fnana.2012.00008 22435053 PMC3303085

[B79] Scheibel A. B. (1977). Sagittal organization of mossy fiber terminal systems in the cerebellum of the rat: a golgi study. Exp. Neurol. 57, 1067–1070. 10.1016/0014-4886(77)90130-3 72676

[B80] Seone A. Apps R. Balbuena E. Herrero L. Liorens J. (2005). Differential effects of trans-crotononitrile and 3-acetylpyridine on inferior olive integrity and behavioural performance in the rat. Eur. J. Neurosci. 22, 880–894. 10.1111/j.1460-9568.2005.04230.x 16115211

[B110] Serapide M. F. Panto M. R. Parenti A. Zappala A. Cicirata F. (2001). Multiple zonal projections of the basilar pontine nuclei to the cerebellar cortex of the rat. J. Comp. Neurol. 430, 471–484. 10.1002/1096-9861(20010219)430:4<471::aid-cne1044>3.0.co;2-g 11169481

[B81] Simpson J. I. (2011). Crossing zones in the vestibulocerebellum: a commentary. Cerebellum 10, 515–522. 10.1007/s12311-011-0305-y 21822546

[B82] Simpson J. L. Graf W. (1985). The selection of reference frames by nature and its investigators. Rev. Oculomot. Res. 1, 3–16. 3940037

[B83] Stanton G. B. (1988). Topographical organization of ascending cerebellar projections from the dentate and interposed nuclei in Macaca mulatta: an antergrade degeneration study. J. Comp. Neurol. 190, 699–731. 10.1002/cne.901900406 6772694

[B84] Stoodley C. J. Scmahmann J. D. (2009). Functional topography in the human cerebellum.: a metaanalysis of neuroimaging studies. Neuroimage 44, 489–501. 10.1016/j.neuroimage.2008.08.039 18835452

[B85] Strick P. L. Dum R. P. Fiez J. A. (2009). Cerebellum and nonmotor function. Ann. Rev. Neurosci. 32, 413–434. 10.1146/annurev.neuro.31.060407.125606 19555291

[B86] Strominger N. L. Truscott T. C. Miller R. A. Royce G. J. (1979). An autoradiographic study of the rubroolivary tract in the rhesus monkey. J. Comp. Neurol. 183, 33–45. 10.1002/cne.901830104 102667

[B87] Sugihara I. Shinoda Y. (2004). Molecular, topographic and functional organization of the cerebellar cortex: a study with combined aldolase C and olivocerebellar labeling. J. Neurosci. 24, 8771–8785. 10.1523/jneurosci.1961-04.2004 15470143 PMC6729951

[B88] Sugihara I. Wu H. S. Shinoda Y. (2001). The entire trajectories of single olivocerebellar axons in the cerebellar cortex and their contribution to cerebellar compartmentalization. J. Neurosci. 21, 7715–7723. 11567061 10.1523/JNEUROSCI.21-19-07715.2001 PMC6762911

[B89] Sultan F. Augath A. Hamodeh S. Murayama Y. Oeltermann A. Rauch A.. (2012). Unravelling cerebellar pathways with high temporal precision targeting motor and extensive sensory and parietal networks. Nat. Commun. 3:924. 10.1038/ncomms1912 22735452

[B90] Teune T. M. van der Burg J. van der Moer J. Voogd J. Ruigrok T. J. H. (2000). Topography of cerebellar nuclear projections to the brain stem in the rat. Prog. Brain Res. 124, 141–172. 10.1016/s0079-6123(00)24014-4 10943123

[B91] Tokuno H. Takada M. Nambu A. Inase M. (1995). Somatotopical projections from the supplementary motor area to the red nucleus in the macaque monkey. Exp. Brain Res. 106, 351–355. 10.1007/BF00241130 8566199

[B92] Van der Steen J. Simpson J. I. Tan J. (1994). Fubctional and anatomoic organization of three-dimensional eye movements in rabbit cerebellar flocculus. J. Neurophysiol. 72, 31–46. 7965015 10.1152/jn.1994.72.1.31

[B93] Van der Want J. J. Vrensen G. F. Voogd J. (1985). Differences in synaptic size in the superficial and deep layers of the molecular layer of the cerebellar cortex of the cat. An electronmicroscopic and autoradiographic study. Anat. Embryol. (Berl) 172, 303–309. 10.1007/bf00318978 4061870

[B94] van Kan P. L. E. Hou J. C. Gibson A. R. (1993). Output organization of intermediate cerebellum of the monkey. J. Neurophysiol. 69, 57–73. 8433134 10.1152/jn.1993.69.1.57

[B95] Voogd J. (1964). The Cerebellum of the Cat. Thesis Leiden. Assen: van Gorcum & Co.

[B96] Voogd J. (1969). “The importance of fiber connections in the comparative anatomy of the mammalian cerebellum,” in Neurobiology of Cerebellar Evolution and Development, ed Llinas R. (Chicago: AMA), 493–514.

[B97] Voogd J. (2012). A note on the definition and the development of cerebellar Purkinje cell zones. Cerebellum 11, 422–425. 10.1007/s12311-012-0367-5 22396330 PMC3359460

[B98] Voogd J. Pardoe J. Ruigrok T. J. Apps R. (2003). The distribution of climbing and mossy fiber collateral branches from the copula pyramidis and the paramedian lobule: congruence of climbing fiber cortical zones and the pattern of zebrin banding within the rat cerebellum. J. Neurosci. 23, 4645–4656. 12805304 10.1523/JNEUROSCI.23-11-04645.2003 PMC6740790

[B99] Voogd J. Ruigrok T. J. (2004). “Cerebellum and precerebellar nuclei,” in The Human Nervous System, eds Paxinos G. Mai J. K. (Amsterdam: Elsevier), 321–392.

[B100] Voogd J. Schraa-Tam C. K. van der Geest J. N. De Zeeuw C. I. (2012). Visuomotor cerebellum in human and nonhuman primates. Cerebellum 11, 392–410. 10.1007/s12311-010-0204-7 20809106 PMC3359447

[B101] Wu H. S. Sugihara I. Shinoda Y. (1999). Projection patterns of single mossy fibers originating from the lateral reticular nucleus in the rat cerebellar cortex and nuclei. J. Comp. Neurol. 411, 97–118. 10.1002/(sici)1096-9861(19990816)411:1<97::aid-cne8>3.0.co;2-o 10404110

[B102] Xiao J. Cerminara N. L. Kotssurovskyy Y. Aoki H. Burroughs A. Wise K. A.. (2014). Systematic regional variaions in Purkinje cell spiking patterns. PLoS One 9:e105633. 10.1371/journal.pone.0105633 25144311 PMC4140808

[B103] Xiong G. Hiramatsu T. Nagao S. (2002). Corticopontocerebellar pathway from the prearcrcuate region to hemispheric lobule VII of the cerebellum: an antegrade and retrograde tracing study in the monkey. Neurosci. Lett. 322, 173–176. 10.1016/s0304-3940(02)00108-8 11897166

[B104] Zhou H. Lin Z. Voges K. Ju C. Gao Z. Bosman L. W. J.. (2014). Cerebellar modules operate at different frequencies. Elife 3:e02536. 10.7554/eLife.02536 24843004 PMC4049173

